# Supplementary material for: Peripheral nerve regeneration following scaffold-free conduit transplant of autologous dermal fibroblasts: a non-randomised safety and feasibility trial
Source: Commun Med (Lond). 2024 Jan 26;4:12. doi: 10.1038/s43856-024-00438-6 (PMC10817910; doi:10.1038/s43856-024-00438-6)
Supplement: Supplementary file 2 — Supplementary Information [file 43856_2024_438_MOESM2_ESM.pdf]

**Supplementary Table 1. Study schedule**

| Protocol: treatment and assessments | Screening | Culture process: Skin biopsy pre | Culture process: Skin biopsy post | Culture process: 1 week after skin biopsy | Treatment: Pre-transplantation (hospitalization), day -1 | Treatment: Transplantation, day 1 | Treatment: Transplantation, day 2 | Treatment: Discharge from hospital, day 4 | Observation: Week 1 | Week 4 | Week 8 | Week 12 | Week 16 | Week 20 | Week 24 | Week 28 | Week 32 | Week 36 | Week 40 | Week 44 | Week 48 | Discontinuation of transplantation | Discontinuation after transplantation |
|---|---|---|---|---|---|---|---|---|---|---|---|---|---|---|---|---|---|---|---|---|---|---|---|
| Permissible range | 30 days within registration | 30 days within confirmation of eligibility | | ±4 days | 7 days within transplanting | 60 ± 21 days after skin biopsy | | | ±4 days | ±7 days | ±7 days | ±7 days | ±7 days | ±7 days | ±7 days | ±7 days | ±7 days | ±7 days | ±7 days | ±7 days | ±28 days | | |
| Informed consent | X | | | | | | | | | | | | | | | | | | | | | | |
| Subject background | X | | | | | | | | | | | | | | | | | | | | | | |
| Interview, medical examination | X | X | | X | X | X | X | X | X | X | X | X | X | X | X | X | X | X | X | X | X | X | X |
| Present complains | X | | | | | | | | | | | | | | | | | | | | | | |
| Inclusion/exclusion criteria | X | | | | | | | | | | | | | | | | | | | | | | |
| Medical history | X | | | | | | | | | | | | | | | | | | | | | | |
| Physical examination*1 | X | X | | X | X | X | X | X | X | | | | | | | | | | | | | | |
| Subjective and objective symptoms | X | X | X | X | X | X | X | X | X | X | X | X | X | X | X | X | X | X | X | X | X | X | X |
| **Clinical tests** | | | | | | | | | | | | | | | | | | | | | | | |
| Haematologic test*2 | X | | | | X | | | | | X | | X | | | X | | | X | | | X | | X |
| Haematologic biochemical test*3 | X | | | | X | | | | | X | | X | | | X | | | X | | | X | | X |
| Coagulation test | X | | | | | | | | | | | | | | | | | | | | | | |
| Infection analysis | X | | | | | | | | | | | | | | | | | | | | | | |
| Urine qualitative analysis | X | | | | | | | | | | | | | | | | | | | | | | |
| Chest X-ray | X | | | | | | | | | | | | | | | | | | | | | | |
| Electrocardiography | X | | | | | | | | | | | | | | | | | | | | | | |
| **Nerve system and functional tests** | | | | | | | | | | | | | | | | | | | | | | | |
| Sensory functional test (SWT) | X | | | | | | | | | X | | X | | | X | | | X | | | X | | X |
| Sensory functional test (s2PD, m2PD) | X | | | | | | | | | X | | X | | | X | | | X | | | X | | X |
| Assessment of upper limb impairment | X | | | | | | | | | X | | X | | | X | | | X | | | X | | X |
| Motor nerve function analysis (MMT) | X** | | | | | | | | | X** | | X** | | | X** | | | X** | | | X** | | X** |
| Pregnancy test | X | | | | | | | | | | | | | | | | | | | | | | |
| Adverse event, failure | | | ← | … | … | … | … | … | … | … | … | … | … | … | … | … | … | … | … | … | → | X | X |
| Information regarding combination treatment | ← | … | … | … | … | … | … | … | … | … | … | … | … | … | … | … | … | … | … | … | → | X | X |

*1: blood pressure, body weight, body temperature
*2: RBC, WBC, PLT, HGB, HCT, Diff, screening: MCV, MCH, MCHC
*3: TP, Alb, AST, ALT, ALP, T-Bil, Cr, BUN, CK, FBS, Na, K, Cl, Ca, CRP
**: Conducted only for eligible individuals
SWT: Semmes–Weinstein monofilament test
s2PD: static 2-point discrimination test; m2PD: moving 2-point discrimination test; MMT: Manual Muscle Test
Abbreviations
RBC: Red Blood Cell; WBC: White Blood Cell; PLT:Platelet; HGB: Hemoglobin; HCT: Hematocrit; Diff: differential white blood count; MCV: Mean corpuscular volume; MCH: Mean corpuscular hemoglobin; MCHC: Mean corpuscular hemoglobin concentration.
TP: Total Protein; Alb: Albumin; AST: Aspartate aminotransferase; ALT: Alanine aminotransferase; ALP: Alkaline phosphatase ; T-Bil: Total Bilirubin; Cr: Creatinine; BUN: Blood Urea Nitrogen; CK: Creatine Kinase; FBS: Fasting Blood Sugar (Glucose); Na: Sodium; K: Potassium; Cl: Chloride; Ca: Calcium; CRP: C-Reactive
