# Supplementary material for: Peripheral nerve regeneration following scaffold-free conduit transplant of autologous dermal fibroblasts: a non-randomised safety and feasibility trial
Source: Commun Med (Lond). 2024 Jan 26;4:12. doi: 10.1038/s43856-024-00438-6 (PMC10817910; doi:10.1038/s43856-024-00438-6)
Supplement: Supplementary file 3 — Description of Additional Supplementary Files [file 43856_2024_438_MOESM3_ESM.pdf]

Description of Additional Supplementary Files

File Name: Supplementary Data 1
Description: Clinical results shown in Figure 3
