# Supplementary material for: Peripheral nerve regeneration following scaffold-free conduit transplant of autologous dermal fibroblasts: a non-randomised safety and feasibility trial
Source: Commun Med (Lond). 2024 Jan 26;4:12. doi: 10.1038/s43856-024-00438-6 (PMC10817910; doi:10.1038/s43856-024-00438-6)
Supplement: Supplementary file 4 — Supplementary Data 1 [file 43856_2024_438_MOESM4_ESM.pdf]

Source data 1. Clinical results

SWMT

	B3CON01	B3CON02	B3CON03	average	stdev
pre TX	6.65	6.65	6.65	6.65	0.00
4W after TX	6.65	4.31	5.46	5.47	0.96
12W after TX	6.65	4.08	3.61	4.78	1.34
24W after TX	4.56	3.61	3.22	3.80	0.56
36W after TX	4.17	3.61	2.44	3.41	0.72
48W after TX	4.08	2.44	2.44	2.99	0.77

s2PD

	B3CON01	B3CON02	B3CON03	average	stdev
pre TX	20	20	20	20.0	0.0
4W after TX	20	15	20	18.3	2.4
12W after TX	20	9	10	13.0	5.0
24W after TX	20	5	6	10.3	6.8
36W after TX	15	3	4	7.3	5.4
48W after TX	10	3	4	5.7	3.1

m2PD

	B3CON01	B3CON02	B3CON03	average	stdev
pre TX	20	20	20	20.0	0.0
4W after TX	20	15	20	18.3	2.4
12W after TX	20	7	7	11.3	6.1
24W after TX	20	3	4	9.0	7.8
36W after TX	15	2	3	6.7	5.9
48W after TX	10	2	3	5.0	3.6

qDASH

	B3CON01	B3CON02	B3CON03	average	stdev
pre TX	84.1	54.5	95.5	78.0	17.3
4W after TX	84.1	4.5	72.7	53.8	35.1
12W after TX	75	4.5	38.6	39.4	28.8
24W after TX	59.1	4.5	27.3	30.3	22.4
36W after TX	68.2	2.3	25	31.8	27.3
48W after TX	63.6	0	27.3	30.3	26.1
