# Supplementary material for: Peripheral nerve regeneration following scaffold-free conduit transplant of autologous dermal fibroblasts: a non-randomised safety and feasibility trial
Source: Commun Med (Lond). 2024 Jan 26;4:12. doi: 10.1038/s43856-024-00438-6 (PMC10817910; doi:10.1038/s43856-024-00438-6)
Supplement: Supplementary file 5 — Reporting Summary [file 43856_2024_438_MOESM5_ESM.pdf]

nature portfolio

Corresponding author(s): Ryosuke Ikeguchi

Last updated by author(s): Jan 11, 2024

Reporting Summary

Nature Portfolio wishes to improve the reproducibility of the work that we publish. This form provides structure for consistency and transparency in reporting. For further information on Nature Portfolio policies, see our Editorial Policies and the Editorial Policy Checklist.

Statistics

For all statistical analyses, confirm that the following items are present in the figure legend, table legend, main text, or Methods section.

n/a	Confirmed	
☐	☒	The exact sample size (*n*) for each experimental group/condition, given as a discrete number and unit of measurement
☐	☒	A statement on whether measurements were taken from distinct samples or whether the same sample was measured repeatedly
☐	☒	The statistical test(s) used AND whether they are one- or two-sided<br>*Only common tests should be described solely by name; describe more complex techniques in the Methods section.*
☐	☒	A description of all covariates tested
☐	☒	A description of any assumptions or corrections, such as tests of normality and adjustment for multiple comparisons
☒	☐	A full description of the statistical parameters including central tendency (e.g. means) or other basic estimates (e.g. regression coefficient) AND variation (e.g. standard deviation) or associated estimates of uncertainty (e.g. confidence intervals)
☒	☐	For null hypothesis testing, the test statistic (e.g. *F*, *t*, *r*) with confidence intervals, effect sizes, degrees of freedom and *P* value noted<br>*Give P values as exact values whenever suitable.*
☒	☐	For Bayesian analysis, information on the choice of priors and Markov chain Monte Carlo settings
☒	☐	For hierarchical and complex designs, identification of the appropriate level for tests and full reporting of outcomes
☒	☐	Estimates of effect sizes (e.g. Cohen's *d*, Pearson's *r*), indicating how they were calculated

Our web collection on statistics for biologists contains articles on many of the points above.

Software and code

Policy information about availability of computer code

Data collection	The study includes no data collection through commercial, open source or custom code.
Data analysis	SAS version 9.4 (SAS Institute, Cary, NC, USA)

For manuscripts utilizing custom algorithms or software that are central to the research but not yet described in published literature, software must be made available to editors and reviewers. We strongly encourage code deposition in a community repository (e.g. GitHub). See the Nature Portfolio guidelines for submitting code & software for further information.

Data

Policy information about availability of data

All manuscripts must include a data availability statement. This statement should provide the following information, where applicable:

- Accession codes, unique identifiers, or web links for publicly available datasets
- A description of any restrictions on data availability
- For clinical datasets or third party data, please ensure that the statement adheres to our policy

The datasets analyzed during the current study available from the corresponding author on reasonable request.

Human research participants

Policy information about studies involving human research participants and Sex and Gender in Research.

Reporting on sex and gender	This study does not include sex- or gender-based analysis.
Population characteristics	See above.
Recruitment	The participants with severed peripheral nerve injuries or defects who desired to participate to this study were recruited.
Ethics oversight	This study was approved by the Institutional Review Board of authors' organization.

Note that full information on the approval of the study protocol must also be provided in the manuscript.

Field-specific reporting

Please select the one below that is the best fit for your research. If you are not sure, read the appropriate sections before making your selection.

☒ Life sciences ☐ Behavioural & social sciences ☐ Ecological, evolutionary & environmental sciences

For a reference copy of the document with all sections, see nature.com/documents/nr-reporting-summary-flat.pdf

Life sciences study design

All studies must disclose on these points even when the disclosure is negative.

Sample size	The sample size was decide with agreement with the Pharmaceuticals and Medical Devices Agency, Japan
Data exclusions	No data was excluded.
Replication	This was a single institution, non-blinded, non-randomised controlled trial. The study cannot make reproducibility.
Randomization	Non-randomised controlled trial
Blinding	Non-blinded trial

Reporting for specific materials, systems and methods

We require information from authors about some types of materials, experimental systems and methods used in many studies. Here, indicate whether each material, system or method listed is relevant to your study. If you are not sure if a list item applies to your research, read the appropriate section before selecting a response.

Materials & experimental systems

n/a	Involved in the study	
☒	☐	Antibodies
☒	☐	Eukaryotic cell lines
☒	☐	Palaeontology and archaeology
☒	☐	Animals and other organisms
☐	☒	Clinical data
☒	☐	Dual use research of concern

Methods

n/a	Involved in the study	
☒	☐	ChIP-seq
☒	☐	Flow cytometry
☒	☐	MRI-based neuroimaging

Clinical data

Policy information about clinical studies

All manuscripts should comply with the ICMJE guidelines for publication of clinical research and a completed CONSORT checklist must be included with all submissions.

Clinical trial registration	the Japan Registry of Clinical Trials (jRCT2053200022, Registered on 1 June 2020)
Study protocol	https://jrct.niph.go.jp/en-latest-detail/jRCT2053200022

March 2021

Data collection	From June. 01, 2020 to November. 30, 2022
Outcomes	Primary outcome;<br>Safety<br>Incidence of adverse events that cannot be ruled out as related to investigational products or transplantation of investigational products, and skin tissue harvesting<br><br>Secondary outcome;<br>Effectiveness<br>1)Sensory functional results by SW test 48 weeks after the surgery<br>2)Sensory functional results by the two-point identification test 48 weeks after the surgery<br>1:Static two-point discrimination<br>2:Dynamic two-point discrimination
